# A Cascaded Model Based on EfficientDet and YOLACT++ for Instance Segmentation of Cow Collar ID Tag in an Image

**DOI:** 10.3390/s21206734

**Published:** 2021-10-11

**Authors:** Kaixuan Zhao, Ruihong Zhang, Jiangtao Ji

**Affiliations:** College of Agricultural Equipment Engineering, Henan University of Science and Technology, Luoyang 471023, China; kx.zhao@haust.edu.cn (K.Z.); rh_zhang@stu.haust.edu.cn (R.Z.)

**Keywords:** cow identification, EfficientDet, YOLACT++, cascaded model, instance segmentation

## Abstract

In recent years, many imaging systems have been developed to monitor the physiological and behavioral status of dairy cows. However, most of these systems do not have the ability to identify individual cows because the systems need to cooperate with radio frequency identification (RFID) to collect information about individual animals. The distance at which RFID can identify a target is limited, and matching the identified targets in a scenario of multitarget images is difficult. To solve the above problems, we constructed a cascaded method based on cascaded deep learning models, to detect and segment a cow collar ID tag in an image. First, EfficientDet-D4 was used to detect the ID tag area of the image, and then, YOLACT++ was used to segment the area of the tag to realize the accurate segmentation of the ID tag when the collar area accounts for a small proportion of the image. In total, 938 and 406 images of cows with collar ID tags, which were collected at Coldstream Research Dairy Farm, University of Kentucky, USA, in August 2016, were used to train and test the two models, respectively. The results showed that the average precision of the EfficientDet-D4 model reached 96.5% when the intersection over union (IoU) was set to 0.5, and the average precision of the YOLACT++ model reached 100% when the IoU was set to 0.75. The overall accuracy of the cascaded model was 96.5%, and the processing time of a single frame image was 1.92 s. The performance of the cascaded model proposed in this paper is better than that of the common instance segmentation models, and it is robust to changes in brightness, deformation, and interference around the tag.

## 1. Introduction

Using machine vision and video surveillance equipment to automatically analyze the behavior of dairy cows, obtain their physiological and health information, and provide data support and decision-making bases for precision breeding and management has gradually become a research hotspot [1,2,3,4]. Machine vision systems have the advantages of no contact, low cost, and low stress [5]. However, the lack of stable and reliable image-based individual identification methods and technologies seriously restricts the promotion and use of these machine vision systems [6].

Radio frequency identification (RFID) is an individual identification method commonly used on large commercial dairy farms. RFID enables the recording of individual information, feeding information [7,8], and milk production [9,10] of dairy cows by reading tags attached to their bodies (usually ear tags) with wireless transmission technology. Compared with traditional methods, the reliability of information and the real-time information acquisition performance of RFID are improved. However, the workload of wearing and maintaining ear tags is substantial, which causes a stress response in the animals [11]. The RFID working performance is affected by label power, iron fences, and electromagnetic environments [12]. Additionally, when there are multiple cows in a recognition scene, individual information from multiple cows cannot be simultaneously obtained. Individual RFID recognition devices add complexity to machine-vision-based intelligent information perception systems. Therefore, scholars have begun to study individual biometrical identification methods based on machine vision, and this recognition is realized mainly through the extraction and classification of the biological characteristics of cows [13]. The muzzle, iris, and face of the head of dairy cows contain various biological information that can be used as recognizable biological characteristics of individual cows. Different algorithms have been used to extract the descriptive features from images of muzzles [14,15], irises [16,17], and faces [18], and machine learning technology is used to classify the feature vectors to achieve individual recognition. However, the acquisition of head images requires a special shooting environment. The shooting angle, the quality of light, and the matching degree of cows all affect the details in the image and can reduce recognition accuracy.

Holstein cows are the most common cows on farms. The black and white patterns on the bodies can be used as biological features to identify individual animals. Zhao and He [19] proposed an individual cow recognition method based on a convolutional neural network. A 48 × 48 matrix from the trunk image of a dairy cow was extracted as a feature value, and a recognition model based on a convolutional neural network was constructed and trained. In the test, 90.55% of the images were correctly identified. Zhao et al. [6] proposed a cow recognition method based on template matching. The feature template library was generated by extracting the trunk image features of all cows, and individual cows were identified by matching their trunk image features with the features in the template library. Okura et al. [11] proposed a method for individual identification of dairy cows based on RGB-D video. The RGB images were used to obtain the texture features of dairy cows, and the depth image videos were used to obtain the features of the cows’ gaits. These two complementary features were used to identify the cows. He et al. [20] proposed an individual identification method based on an improved YOLO v3 model. Images of cow backs were obtained with video frame decomposition technology, and a recognition model of optimizing anchors and improving network structure was constructed based on the Gaussian YOLO v3 algorithm. Yukun et al. [21] obtained images of the backs of dairy cows with moving cameras. While constructing an automatic system for scoring cow body condition, these authors established an individual recognition model of dairy cows based on a YOLO model and a convolution neural network. Side-view or top-view images of walking dairy cows are easy to obtain. Generally, a video or image acquisition device can be placed in the vicinity of dairy cows during feeding, drinking, or milking, and camera focus can be adapted to different recognition distances. However, this method is only applicable to Holstein cows with black and white patterns, which does not solve the problem of identifying cows with uniform colors on their bodies. In addition, the output dimension of the network corresponds to the number of cows in the herd. When the number of cows increases, the scale of the network increases exponentially. Once new cows join the herd, the entire network needs to be retrained.

An individual dairy cow identification model that functions through the detection and recognition of the ID number on their collar tag requires simple description features and a small network scale compared to biometric identification. The tagging and maintenance processes of this method impose a lower workload than RFID and do not affect the welfare of the dairy cows compared. Specifically, the ID tag worn on the cow’s neck is first located, and then the ID numbers on the tag are recognized to identify the dairy cow. Zhang et al. [22] proposed a method of cow individual identification based on collar ID tags. This method first locates the tag by cascade detector combined with multi-angle detection, and then performs character segmentation and character recognition on the tag image. However, this location method cannot well-adapt to distortion deformation of ID tags. Zin et al. [23] proposed a tracking system for individual cows using visual ear tag analysis. First, the head and ear tag are detected. Then, the ear tag is recognized by finding the four-digit area, digit segmentation and digit recognition. However, the wearing process of ear tags requires punching a hole in the ear of cows, which can easily cause a stress response and affects the welfare of cows. The cascaded instance segmentation method we propose can adapt to the various deformations of ID tags, and its wearing process does not have much of an effect on the physiology and psychology of dairy cows. A comparison of different identification methods for cows is provided in Table A1.

The detection of ID tags is the first and key step to identifying individual cows, and its results directly affect the subsequent character recognition accuracy. If the ID tag is accurately segmented according to its contour, the digital recognition task becomes similar to license plate character recognition. According to existing research, it has achieved high recognition accuracy [24,25]. At present, few studies have been conducted on cow collar tag detection, but there are many studies on and applications for license plate detection. Xie et al. [26] proposed a multidirectional license plate detection framework based on CNN, which predicts the rectangular box and corresponding rotation angle to the license plate. This method can solve the problem of license plate rotation in a plane, but it cannot accommodate the tilt of the plate in three-dimensional space caused by the shooting angle. Xu et al. [27] proposed a method for locating irregular quadrilateral license plates. The proposed algorithm has two prediction branches: one is to predict the bounding box containing the license plate area and the other is to predict four groups of vertex offset values corresponding to the four bounding box corners, so as to get the vertex of irregular quadrilateral. This method is implemented based on YOLOv3 that extends the output dimensions. Kim et al. [28] proposed a two-step license plate location method that first detects the vehicle area and then locates the license plate in each vehicle area. This method can quickly filter out the complex background in an image. The license plate that is detected by these methods is a rigid object, but the four digital blocks on the ID tag we aimed to detect are attached to a flexible neck ring (to reduce the foreign body sensation experienced by the cow).

The flexible collar ID tag detection task is required to solve the following two key problems: First, in a side-looking image of a cow walking, the cow is in a continuous state of activity, so the tag is rotated and distorted in different planes, causing different degrees of deformation. Second, when the cow is far from the image acquisition equipment, the pixel area of the tag is relatively small, which causes difficulty in accurate detection. Therefore, we constructed a cascaded model for instance segmentation of the targets. First, the EfficientDet-D4 [29] model is used to detect the bounding box surrounding the ID tag, which effectively filters out most of background in the image and makes the segmentation task more targeted. Then, the image in the bounding box is sent to the YOLACT++ [30] model, and the ID tag is accurately segmented according to its contour to solve the tag deformation problem.

To accurately segment collar ID tags of cows, we conducted the following work: (1) To address the detection and recognition of a cow collar ID tag, we propose a high-precision cascaded model based on EfficientDet and YOLACT++ for instance segmentation, which overcomes the detection difficulty caused by the small area and large deformation of the tag. (2) We tested the performance of EfficientDet-D0–D5 model in the ID tag detection task, and analyzed the ability of different models to detect small targets. (3) The YOLACT++ model with different backbone networks (ResNet50/ResNet101) and different numbers of prototype masks was used to segment ID tags, and the effects of different parameters on the accuracy and speed of a single target segmentation task were analyzed. (4) The common two-stage segmentation models Mask RCNN [31], Mask Scoring RCNN [32], and one-stage instance segmentation model Solov2 [33] were used to segment ID tags, and the accuracy and speed of our proposed method and the above methods were compared. (5) The robustness of the cascaded instance segmentation model to changes in area, ID tag deformation, and brightness was analyzed.

The main contributions of this paper can be described as follows:

(I) A cascaded model is proposed based on EfficientDet and YOLACT++ to accurately detect and segment small targets in images.
(II) The structure and parameters of the model are optimized to improve the detection accuracy and efficiency.

## 2. Materials and Methods

### 2.1. Data Acquisition

**Figure 1:**
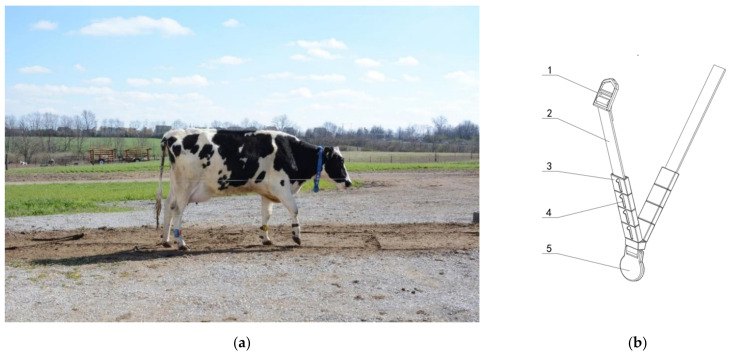
(**a**) An original image. (**b**) A schematic diagram of a neck collar: 1, buckle; 2, neck band; 3, character block; 4, digital label; 5, weight.

Experimental images were collected at Coldstream Research Dairy Farm, University of Kentucky, USA, in August 2016, and the subjects were Holstein cows during lactation. When a cow returned to her shed after milking, she passed through a flat straight passage that had four electric fences (two before and after each) to limit the active area of the cow, and the width of the passage was 2 m. A Nikon D5200 camera was mounted on a tripod 3.5–5 m from the passage and 1.5 m from the ground. The camera used a 35 mm lens and was set to ISO 400, autoexposure, and autofocus. As cows passed through the camera’s field of view, it continuously captured pictures at fixed time intervals. The resolution of the images was 6000 (horizontal) × 4000 (vertical) pixels. Images were captured from 16:00 to 18:00 on sunny days and was performed under natural light. The images were stored in the camera’s local memory card. One of the original images is shown in Figure 1a. The cows were all wearing collars, as shown in Figure 1b. Each collar contained four square blue plastic blocks with white numbers; the four-digit numbers were the only identity labels of the cows.

To verify the background robustness of the cascaded model, we collected images of dairy cows wearing collar ID tags in the feeding bank at Sheng Sheng Farm, in Luoyang, Henan province, China. The images were captured from 9:30 to 11:30 under natural light on 16 September 2021. A cellphone (Xiaomi 10, Xiaomi Inc., Beijing, China) was used for hand-held shooting. The camera was set to autofocus and autoexposure mode. Images were captured from different angles when dairy cows were fixed on fences. A total of 200 images were captured, where 20 cows were involved and four different collar IDs were used. The resolution of the images was 5792 (horizontal) × 4344 (vertical) pixels.

The images were screened to exclude those with no cow or those that were overexposed, leaving 1344 images for the experiment. Due to the different moving speeds of the cows through the field of view, the number of samples of the individuals differed. A total of 670 images of 36 cows were randomly selected as the training set, which included 788 tags. A total of 268 images of 16 cows were randomly selected as the validation set, which included 321 tags. The 406 images of the remaining 26 cows were used as the test set, which included 492 tags. The ratio of images in the training set, validation set, and test set was approximately 5:2:3, and there was no cross-duplication between individuals in different data sets. The training set was used to fit the ID tag detection and segmentation model. The validation set was used to preliminarily evaluate the model to adjust its hyperparameters. The test set was used to evaluate the generalization ability of the final model.

### 2.2. Data Labelling

Labelme software (https://github.com/CSAILVision/LabelMeAnnotationTool (accessed on 22 October 2020) was used to annotate the data and build data sets in COCO format. Because the activities of the cows led to different degrees of deformation of their tags, the polygon mode was selected to label the target in an image. For the cascaded instance segmentation model in this paper, two steps of image annotation were required. Step (1): The tags in the original image were labelled to train and test the ID tag detection model. Step (2): The detection model trained in step (1) was used to detect ID tags in the training set and crop the detected bounding boxes. The cropped images were labelled and taken as the training set for the segmentation model. We performed the same for the validation set and test set of the detection model to obtain the validation set and test set of the segmentation model, respectively. Because the resolution of the original image was too high (4000 × 6000 pixels), the memory requirement of the model training was very high, so the images of the training set and the validation set were compressed to 1200 × 800 pixels when training the ID tag detection model.

### 2.3. Cascaded Model for Instance Segmentation

To solve the problem of the small area and the deformation of the cow collar ID tag in the images, a cascaded detection method was developed in this study. First, the detection model was used to detect the ID tag, and the image in the bounding box surrounding the ID tag was cropped as the input to the segmentation model. Then, the ID tag was accurately segmented according to its contour using the instance segmentation model. For the detection model in the first step, since the area of the target contained a small portion of the whole image, the feature extraction network was required to obtain both high-level semantic information and low-level spatial information. We wanted the model to allow the input image resolution to be as large as possible to retain more feature information. EfficientDet is a scalable model architecture for object detection based on EfficientNet. EfficientDet-D0–D7 were obtained by the composite scaling of each part of the detection network. This composite scaling method enabled us to balance accuracy and speed and to choose a better model. The BiFPN structure in the EfficientDet model enabled the network to obtain rich semantic and spatial information about the target through the upsampling, downsampling, and weighted fusion of different feature layers. Therefore, we chose EfficientDet as the ID tag detection model and tested the performance of different EfficientDet models to identify and select the optimal model.

For the ID tag segmentation task, the segmentation result was the final result, which directly affected the accuracy of the subsequent character recognition. Therefore, we hoped that the mask along the edge of the tag could completely contain all the ID numbers and did not contain redundant background. Because the image to be segmented was relatively small and the target area generally occupied more than 1/2 of the whole image, the difficulty of segmentation was low, and the fully convolutional network could efficiently segment the ID tags. Therefore, we chose the real-time instance segmentation model YOLACT++ based on a fully convolutional network to complete the tag segmentation task.

#### 2.3.1. EfficientDet Detection Model

**Figure 2:**
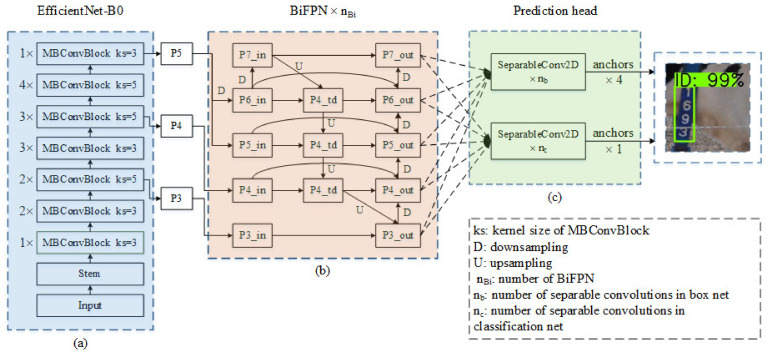
The structure of EfficientDet. (**a**) EfficientNet-B0, (**b**) BiFPN, (**c**) prediction head.

EfficientDet uses EfficientNet as its backbone to extract feature maps. EfficientNet obtains EfficientNet-B0–B7 by scaling the baseline model while adjusting the depth, width, and resolution of the input image. As the baseline, EfficientNet-B0 is composed of 1 stem and 7 blocks, as shown in Figure 2a. The stem structure functions to adjust the number of channels through convolution. The block includes several mobile inverted bottleneck convolution (MBConv) block modules. The design concept of the MBConv block modules involves inverted residuals and ResNet. First, a 1 × 1 convolution is performed to upgrade the dimension of the feature maps and a 3 × 3 or 5 × 5 depthwise separable convolution is performed, then a simple attention mechanism is added after this structure. Finally, 1 × 1 convolution is used to reduce the dimensionality of the feature maps, which are connected to the input side to form a residual structure. The channel attention mechanism effectively reduces the redundant channel feature information in the image, accelerates the network training speed, and reduces the memory required for training. Based on EfficientNet-B0, EfficientNet-B1–B7 are obtained by changing the width coefficient, depth coefficient, and input image size.

Simply, BiFPN is an enhanced version of FPN. The feature extraction process of BiFPN is shown in Figure 2b. BiFPN mainly includes two parts: the first is feature upsampling and feature weighted fusion; the second is feature downsampling and feature-weighted fusion. After downsampling and adjusting the number of channels, the feature maps extracted by EfficientNet are used as the input to BiFPN. First, upsampling and stacking of input features are performed, and then downsampling and stacking are performed. In the next BiFPN, the feature layers of the previous stage are used as the input, and up and downsampling and feature fusion are carried out again. This feature extraction method of upper and lower circulation sampling and weighted fusion retains the spatial information of the ID tag and obtains semantic information. From EfficientDet-D0 to EfficientDet-D7, BiFPN has an increasing number of cycles, which means that the depth of the network increases and the extracted feature information is richer. However, with the deepening of the network, the speed of training and reasoning is reduced.

The prediction head consists of two parts: the classification network and the prediction box regression network. The former assesses the category of the target, and the latter regresses the location of the target, as shown in Figure 2c. Before prediction, anchors are generated on the feature layers extracted by BiFPN. Through repeated separable convolutions, the classification branch and the prediction box regression branch generate 1 category parameter and 4 position adjustment parameters for each anchor, and finally obtain the location of the prediction box and the category of the target in the prediction box. From EfficientDet-D0 to EfficientDet-D7, the classification branch and the prediction box regression branch have different depths. When the EfficientDet head uses more separable convolutions, it may be less sensitive to small targets while acquiring deep semantic information.

Compared with other EfficientDet network structures, the number of parameters of EfficientDet-D6 and EfficientDet-D7 is significantly larger. Considering the image resolution and detection efficiency, we did not consider the use of EfficientDet-D6 or EfficientDet-D7 in the ID tag detection task.

#### 2.3.2. YOLACT++ Segmentation Model

In the YOLACT++ instance segmentation model [30], a series of prototype masks and mask coefficients are generated by a fully convolutional network and fully connected layers, respectively, and the final mask is obtained by a linear combination of the two. As a one-stage model, YOLACT++ also adds a fast mask rescoring network to improve the segmentation accuracy of the mask so the model has excellent detection speed and high segmentation accuracy.

**Figure 3:**
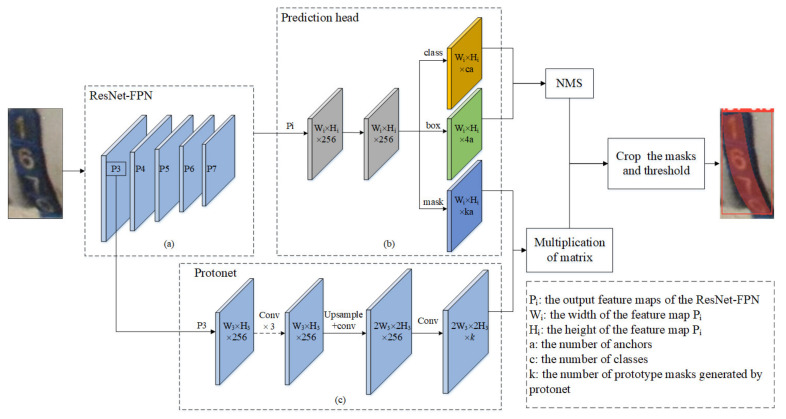
The structure of YOLACT++. (**a**) ResNet-FPN, (**b**) prediction head, (**c**) protonet.

The YOLACT++ model uses ResNet as its backbone and FPN to construct feature maps P3, P4, P5, P6, and P7 with different sizes and advanced semantic information, as shown in Figure 3a. To adapt to the different scales and deformations of the target, a deformable convolutional network (DCN) is introduced into ResNet. The prototype generation branch (Protonet) takes the P3 layer of FPN (feature pyramid net) as its input (Figure 3c) because the P3 layer, as the deep backbone feature, has high resolution and can produce high-quality masks. Protonet is a fully convolutional network (FCN) composed of 3 × 3 and 1 × 1 convolution layers. Protonet predicts *k* prototype masks for the image, and all the final predicted masks are the linear combination of these *k* prototype masks. The prediction head takes the five feature maps (*Pi*) output by FPN as its input and uses the fully connected layer to generate three branches. One branch is used to predict the confidence of the target belonging to *c* categories, the second branch is used to predict the four position regression parameters of the bounding box, and the third branch is used to predict *k* mask coefficients (*k* corresponds to the number of prototype masks), as shown in Figure 3b. Then, non-maximum suppression (NMS) is carried out according to the predicted bounding box and the corresponding category confidence. The linear combinations of prototype masks and corresponding mask coefficients are the results of instance segmentation. These operations can be efficiently implemented using a single matrix multiplication and sigmoid:

(1) $$M=\;\sigma \left(PC^{T}\right)$$

where *P* is an h×w×k matrix of prototype masks and *C* is an n×k matrix of mask coefficients for *n* instances that survive NMS and score thresholds. Finally, the masks are cropped with the predicted bounding box.

### 2.4. Training Platform and Parameter Settings

#### 2.4.1. Training Platform

The software environment of our experimental platform was an Ubuntu 18.04 LTS 64 bit system. The programming language was Python 3.7. CUDA10.1 and cuDNN 7.6.5 were used as the parallel computing architecture of the deep neural network and GPU acceleration library. We selected Pytorch 1.4 as the deep learning framework. The GPU was a NVIDIA GeForce GTX 1080Ti, and the memory was 11 GB. The CPU had a 3.50 GHz Intel(R) Core(TM) i7-7800X processor, and its working memory was 32 GB.

#### 2.4.2. Training Parameters of EfficientDet and YOLACT++

First, the training set and validation set constructed in step (1) of Section 2.2 were used to train the ID tag detection model EfficientDet. AdamW was selected as the optimizer for model training, and the batch size was set to 2. The initial learning rate was set to 1 × 10^−3^. If the loss of the validation set was less than 0.1 in three epochs, the learning rate would have become 0.1 times that of the original. The weight decay coefficient and momentum coefficient were set to 1 × 10^−4^ and 0.9, respectively. The maximum number of iterations of all models was 1 × 10^4^. According to the statistics of the tag size in the scaled image, the anchor sizes were determined to be 4, 8, 16, 32, and 64. The K-means clustering algorithm was used to calculate the anchor ratios suitable for our dataset, which were (0.7, 1.4), (1.0, 1.0), and (1.4, 0.7). The same training environment and training parameters were used to train Efficient-D0–D5 based on the pretraining model. After training, the performance of different EfficientDet models was evaluated with test sets.

The training set and validation set constructed in step (2) of Section 2.2 were used to train the ID tag segmentation model YOLACT++. SGD was selected as the optimizer for model training, and the batch size was set to 4. The initial learning rate was set to 1 × 10^−4^. The weight decay coefficient and the momentum coefficient were set to 1 × 10^−4^ and 0.9, respectively. In the training process, the maximum number of iterations of the model was 1 × 10^4^. ResNet50 and ResNet101 were selected as the backbone of YOLACT++ for training to compare the effects of different backbones on the accuracy and speed of the ID tag segmentation model. To study the influence of the generated prototype masks number *k* on the segmentation effect and speed of a single target, the YOLACT++ models were trained with *k* = 4, 16, and 32.

### 2.5. Precision Evaluation Index of Model

In this study, COCO detection evaluation indexes were used to evaluate the precision of the model. The intersection over union (IoU) is a value used to measure the degree of overlap between a prediction box and a groundtruth box, and its formula is:

(2) $$\mathrm{IoU}=\frac{S_{p}\cap S_{g}}{S_{p}\cup S_{g}}$$

where *S_p_* represents the area of the predicted bounding box and *S_g_* represents the area of the groundtruth bounding box. The IoU threshold is used to determine whether the content in the prediction box is a positive sample.

For the target detection model, the commonly used evaluation indices are precision *(P*) and recall (*R*), and their calculation formulas are:

(3) $$P=\frac{TP}{TP+FP}$$

(4) $$R=\frac{TP}{TP+FN}$$

where *TP* represents the number of correctly predicted targets; *FP* represents the number of falsely predicted targets, that is, the background is mistaken for a positive sample; *FN* represents the number of missed targets, that is, a positive sample is mistaken as the background. Confidence is an important indicator in target detection algorithms. For each prediction box, a confidence value was generated, indicating the credibility of the prediction box. Different combinations of *P* and *R* were obtained by setting different confidence thresholds. Taking *P* and *R* as vertical and horizontal coordinates, respectively, the *PR* curve could be drawn. When the IoU threshold was set to 0.5, the area under the PR curve was AP^IoU = 0.50^ (AP50). When the IoU threshold was set to 0.75, the area under the PR curve was AP^IoU = 0.75^ (AP75). AP was averaged over multiple intersection over union (IoU) values. Specifically, we used 10 IoU thresholds of 0.50:0.05:0.95. The average of multiple IoU thresholds more comprehensively reflects the performance of the model.

From the statistics of the test results, for the first step of the ID tag detection task, only when the IoU of the prediction and groundtruth bounding box was greater than 0.5 could the prediction box contain all the numbers on a tag. Therefore, the AP^IoU = 0.50^ (AP50) and AP of the detected bounding boxes were selected as the evaluation indices of the accuracy of the tag detection model. For the second step of the ID tag segmentation task, only when the IoU of the prediction and groundtruth mask was greater than 0.75 could the prediction mask contain all the numbers on a tag without background. Therefore, the AP^IoU = 0.75^ (AP75) and AP of the segmented masks were selected as the evaluation indices of the accuracy of the tag segmentation model. For the proposed cascaded instance segmentation method, we multiplied the AP50 of the detection model and the AP75 of the segmentation model to obtain the final accuracy of the ID tag detection model.

## 3. Results

### 3.1. Training and Testing of EfficientDet

During EfficientDet training, in the first 1000 iterations, the loss decreased rapidly. In 1000–6000 iterations, the loss had no obvious convergence trace but continuously oscillated. After 6000 iterations, due to the reduction in the learning rate, the loss started to converge again and finally reached a stable state. Therefore, for EfficientDet, reducing the learning rate at the late training stage effectively inhibited the loss oscillation of the model and accelerated the convergence rate. From EfficientDet-D0 to EfficientDet-D5, the training time gradually increased from the initial 3 h to 43 h, indicating that the complexity of the network structure significantly affected the training time of the model.

**Figure 4:**
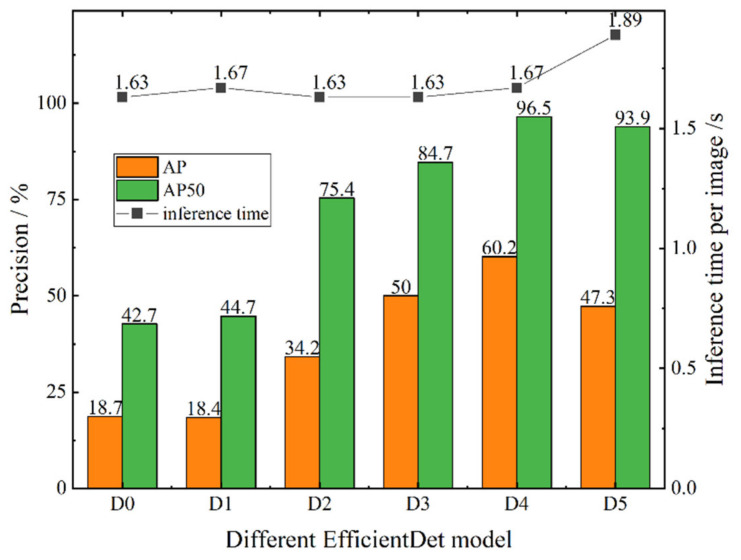
The precision and efficiency of EfficientDet-D0–D5. D0–D5 represent EfficientDet-D0–EfficientDet-D5, respectively.

To test the performance of different EfficientDet models in the ID tag detection task, the original images (6000 × 4000 pixels) in the test set, which were constructed in step (1) in Section 2.2, were input to the trained Efficient-D0–D5 models for detection. According to the detection results, we aimed to find the best EfficientDet model that achieved a balance between accuracy and speed. The AP^IoU = 0.50^ (AP50) and AP of the detected bounding boxes and inference time per image were used as he evaluation indices. The test results are shown in Figure 4.

As shown in Figure 4, from EfficientDet-D0 to EfficientDet-D4, the accuracy increased, indicating that increased network depth and multiple BiFPN cycles significantly improved the extraction and expression of image features, and the reasoning time for a single image did not significantly increase. The main factor affecting the reasoning speed of different EfficientDets was the complexity of the model. Although EfficientDet-D0–D4 had different complexities, their parameters were within 5–20 million. For our ID tag detection task, these differences had less influence on the reasoning speed than the high resolution of the image. Thus, the reasoning time of the EfficientDet-D0–D4 models for a single image had no obvious change.

Although EfficientDet-D5 has a wider and deeper network than EfficientDet-D4, its accuracy in the tag detection task was lower than that of EfficientDet-D4. This shows that for our small target detection task, the spatial information of small targets gradually reduced when the network reached a certain depth, which led to a decrease in detection accuracy. However, the number of parameters of the EfficientDet-D5 model was 30 million, which is approximately 1.5 times that of EfficientDet-D4, so its inference time was longer than that of the previous model. Therefore, we finally adopted the EfficientDet-D4 model with its high accuracy and efficiency as the ID tags detection model.

**Figure 5:**
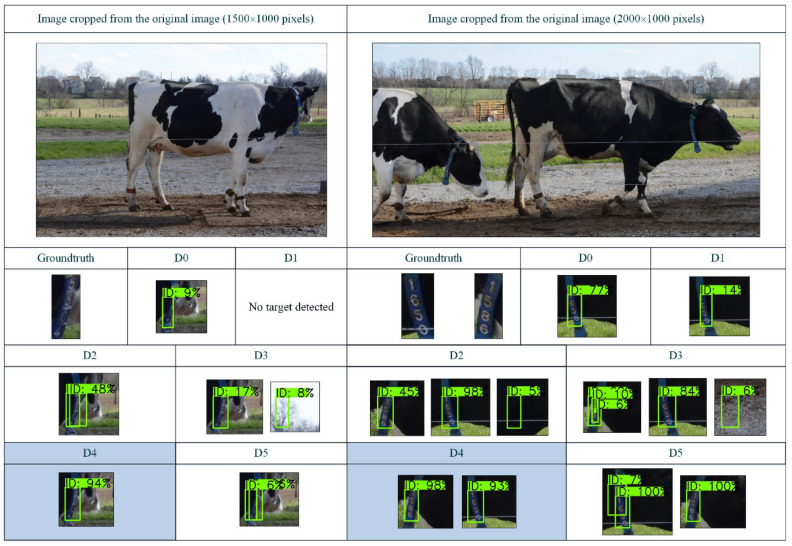
Some of detection results of EfficientDet-D0–D5. Groundtruth represents the true bounding box in the image to be detected; D0–D5 represent EfficientDet-D0–EfficientDet-D5, respectively; the green boxes in the detection image represent the prediction results of the model; and ID represents the class of detected targets. For our ID tag detection task, ID is the only class. The number behind ID represents the confidence of the corresponding detection box, and the unit is % (not shown in some black background images).

Figure 5 shows the detection results of EfficientDet-D0–D5 for some of the images in the test set. Due to the high resolution, only the image content related to the prediction box is cropped.

Figure 5 shows that for EfficientDet-D0 and EfficientDet-D1, problems of missing targets and inaccurate location often occurred, indicating that the shallow network structure could not effectively extract the features of small targets in the image. For EfficientDet-D2 and EfficientDet-D3, there were few missed targets but many false detections. This indicates that improvement in the network depth, width, and input image resolution increased the ability to extract features from small targets, but semantic information sufficient to accurately classify anchors was not extracted. For EfficientDet-D4, the model could not only accurately classify and locate small targets but also had higher confidence in correct classification than the previous model, which accurately and efficiently completed the ID tags detection task. The confidence of the detection boxes of EfficientDet-D5 was high, but there were false samples near the target. This shows that high-level semantic information could correctly classify anchors when the network depth increased, but the low-level spatial information of small targets decreased, resulting in false detection boxes near targets. Thus, for small target detection tasks, reasonable network depth and width are the keys to simultaneously obtaining accurate semantic information and complete position information.

### 3.2. Training and Testing of YOLACT++

During training, the model converged rapidly in the first 500 iterations. From 500 to 6000 iterations, although it stabilized overall, there were still some large loss values. The losses stabilized after the 6000th iteration. The model with the ResNet101 backbone had a slightly longer training time than the model with the ResNet50 backbone. The greater the *k* value, the longer the training time. Compared with the *k* value, the backbone had more influence on the training time.

**Figure 6:**
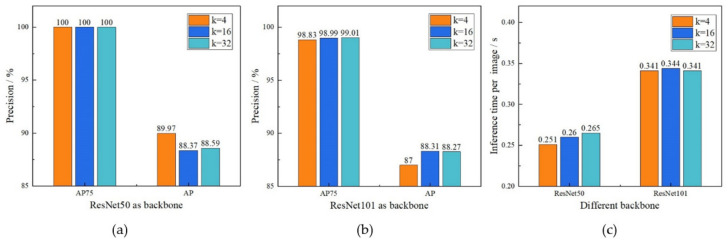
The precision and efficiency of YOLACT++. (**a**) The detection accuracy of the model with ResNet50 as backbone. (**b**) The detection accuracy of the model with ResNet101 as backbone. (**c**) The detection speed of the model with different parameters.

To study the influence of different parameters on the accuracy and detection speed of the ID tag segmentation model, after the training was completed, the images of the test set constructed in step (2) in Section 2.2 were input to the YOLACT++ models with different parameters for segmentation. The AP^IoU = 0.75^ (AP75) and AP of the segmented masks and detection speed were used as test indices. The test results are shown in Figure 6.

As shown in Figure 6a,b, the accuracy index AP75 of the models with different parameters reached nearly or exactly 100%, and the detection time of a single image was 0.25–0.34 s, indicating that the YOLACT++ model could quickly and accurately segment the ID tag through a linear combination of the prototype masks and the mask coefficients. The accuracy of the YOLACT++ model using ResNet50 as the feature extraction network was higher than when using ResNet101 as shown in Figure 6a,b. This result indicates that for simple segmentation tasks, the depth of ResNet50 was sufficient to extract the features of the target in the image. When ResNet50 was used as the backbone, reducing the number of prototype masks generated slightly improved the segmentation accuracy. This shows that for single-target segmentation, due to the reduced background in the image, too many protomasks will interfere with accurate segmentation of a tag.

**Figure 7:**
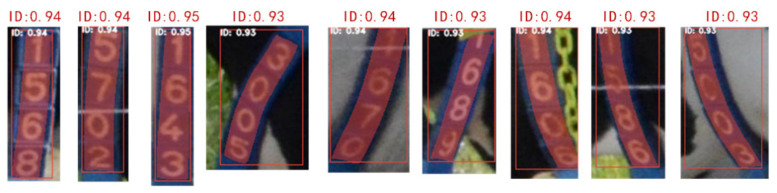
Some of segmentation results of YOLACT++. ID represents the identified class name, and the number after ID represents the confidence for the predicted mask.

In terms of detection speed, the speed of the YOLACT++ model with ResNet50 as its backbone was higher than that of ResNet101, as shown in Figure 6c. When the backbone of YOLACT++ was ResNet50, reducing the *k* value slightly improved the detection speed. However, when the backbone of YOLACT++ was ResNet101, reducing the *k* value had little effect on the detection speed. This result indicates that compared with the k value, the backbone had a greater impact on the detection speed. According to the test results, we finally decided to use ResNet50 as the backbone for feature extraction and chose to generate four prototype masks. As a result, the overall accuracy of the cascaded model based on EfficientDet-D4 and YOLACT++ was 96.5%, and the total detection time for a single image was 1.92 s. Figure 7 shows some test results for the YOLACT++ model. The predicted masks cleanly surrounded the number on the tag with relatively high confidence. The result had good robustness to the rotation of the tag, the change in brightness, and the interference around the label.

## 4. Discussion

### 4.1. Comparison with Common Instance Segmentation Model

The proposed cascaded detection model was compared with the common two-stage models Mask RCNN, Mask Scoring RCNN, and the one-stage model SOLOv2. Mask RCNN and Mask Scoring RCNN are two-stage detection models based on a region proposal network. The detection accuracy of these algorithms is high, but their detection speed is slow. SOLOv2 is a one-stage detection model based on anchor box regression. The detection accuracy of this algorithm is slightly low, but its speed is fast. We used the same training set, validation set, and test set to train and test the accuracy and detection time of different models in the same operating environment. The results are shown in Table 1.

The overall accuracy of our proposed cascaded instance segmentation model is 96.5%, and its detection time for a single image is 1.92 s. The two-stage instance segmentation model Mask RCNN accurately locates and segments most ID tags with high accuracy, and its segmentation index AP75 is 85.3%, but its detection time for a single image is 2.63 s, which is slightly longer. Mask Scoring RCNN with a Re-score branch performs worse than Mask RCNN in our ID tag segmentation task, and its segmentation index AP75 is 58.2%. The detection time per image is 3.39 s, which is longer than that of Mask RCNN. As a one-stage instance segmentation model, SOLOv2 has a short detection time of 1.21 s. However, its segmented mask is rough along the edge of the tag but tortuous, so its segmentation accuracy is low, at 18.5%. In most cases, the masks with tortuous contours contain some background outside the tags.

**Figure 8:**
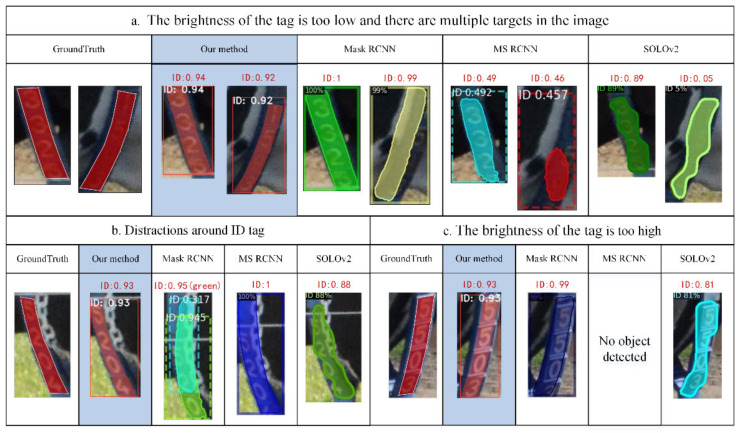
Some of the segmentation results of cascaded model and other models. The ID above the detection results and the number after the ID represent the class and the confidence of the prediction mask, respectively. (**a**–**c**) in the figure correspond to three difficult situations. (**a**) The brightness of the tag is too low and there are multiple targets in the image. (**b**) Distractions around ID tag. (**c**) The brightness is too high.

The detection results for some images are shown in Figure 8. Three situations with high segmentation difficulty are depicted in the figure. The first is when the brightness of the tag is too low and there are multiple targets in the image. The second is when interference around the tag has similar characteristics to characters (such as white chains). Third, when the brightness of the tag is too high, the character block borders are also displayed. In the above three cases, our method accurately segments the ID tag from the complex background. However, other models are prone to location offsets; missing some characters, including redundant backgrounds and even being unable to detect the tag. Therefore, compared with existing two- and one-stage segmentation models, our proposed cascaded instance segmentation method achieves high-precision ID tag segmentation in complex environments, which has strong robustness and solves the problem of detection difficulty caused by the small area and large deformation of the tag.

### 4.2. Deformation and Brightness Robustness

**Figure 9:**
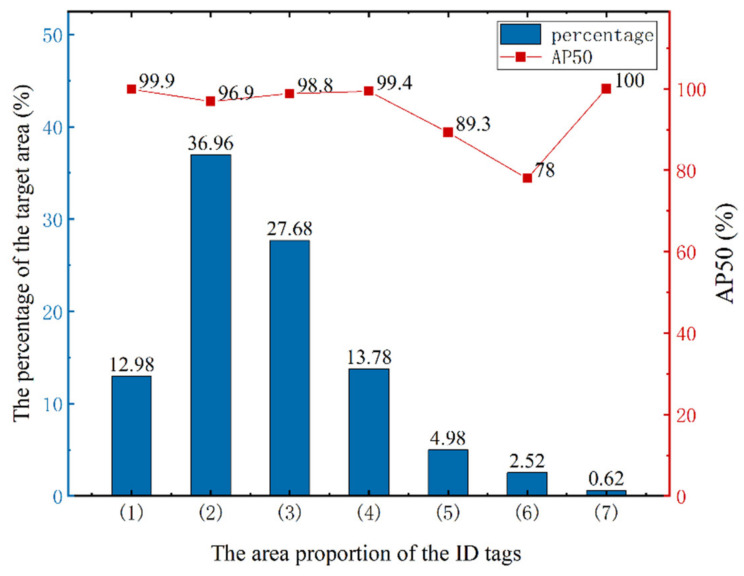
The precision of ID tags with different areas. The abscissa represents the proportion of the bounding box area of the ID tag to the whole image; (**1**) to (**7**) represent seven intervals from 0.02% to 0.09% in increments of 0.01%.

To analyze the performance in detecting targets with different areas, we quantified the results of detecting ID tags with different areas with EfficientDet-D4, as shown in Figure 9. As seen from Figure 9, the proportion of the ID tag area to the whole image was only 0.02–0.09%, which is representative of a small target that was difficult to detect, but the model still achieved a high detection rate. By observing the tags of different areas, we found that the rotation and distortion of the tag were the main reasons for the change in area, and the distance between the cow and the image acquisition equipment was the secondary reason. The larger the area of the ID tag, the larger the deformation of the tag. The detection accuracy of ID tags in intervals (5) and (6) was low. There were two main reasons: (1) the total number of samples in these two intervals was small, so even a small amount of false detection had a relatively large impact on the results; (2) the deformation of the tag led to an increase in false detection boxes that overlapped with the target but did not fully contain the numbers on the tag. There were only three ID tags in interval (7), which had fewer samples. When drawing its *P*–*R* curve, the accuracy and recall rate were both 100% with the confidence threshold set to 0.7, so its AP50 was 100%.

**Figure 10:**
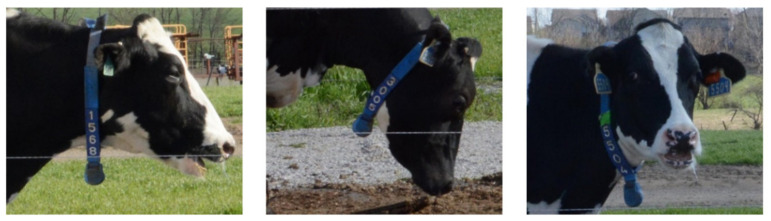
The three types of deformation of ID tags: (**a**) slight rotation or distortion; (**b**) rotation; (**c**) rotation and distortion.

To analyze whether the model is robust to different types of deformation, we divided the deformation of the ID tag into three types (Figure 10): (1) the cow was walking slowly or static, and the tag was only rotated or slightly distorted; (2) the cow was walking quickly or had a lowered head, and the tag was rotated; (3) the cow’s head was twisted, and the tag was both rotated and distorted. The detection results of EfficientDet-D4 for ID tags with different types of deformation were statistically analyzed (Table 2). Table 2 shows that the accuracy was the highest when the tag only slightly rotated or distorted. The accuracy was lower when the tag was rotated. When the tag was both rotated and distorted, the accuracy was the lowest. However, rotation and distortion only reduced the accuracy by 2.9%. Therefore, regardless of the state of the ID tag, the model achieves a high detection rate and has high robustness to different types of deformation.

**Figure 11:**
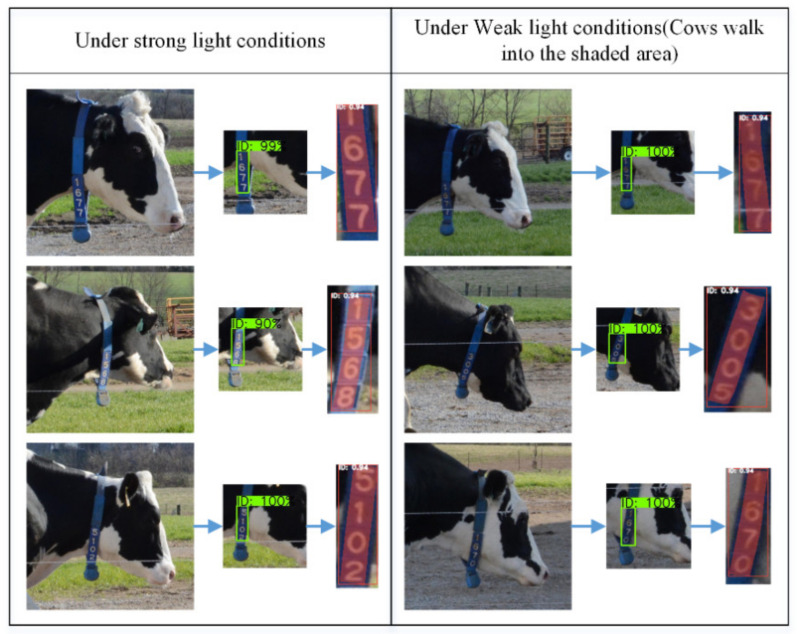
Some detection results under different light conditions.

The cascaded instance segmentation method proposed in this paper can also adapt to the variant brightness of the target. Figure 11 shows the detection results for some ID tags under different light conditions.

### 4.3. Background Robustness

**Figure 12:**
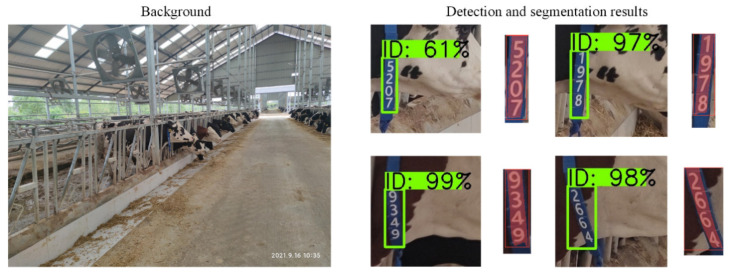
Detection and segmentation results with different backgrounds.

Without retraining the model, the dairy cows’ images collected at Sheng Sheng Farm were passed through the cascaded model for detection and segmentation. The results showed that the AP50 of EfficientDet-D4 model is 94.1%, and the AP75 of YOLACT++ model is 100%. Some test results are shown in Figure 12. Even in different scenarios, the model has achieved high accuracy. Therefore, we concluded that the constructed and trained cascaded instance segmentation model has strong robustness with different backgrounds and has promising application prospects.

### 4.4. Analysis of False and Missed Detections

**Figure 13:**
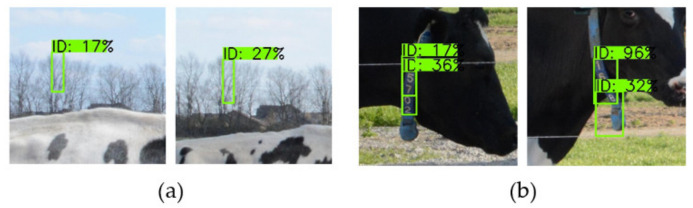
Some of the false detection results. (**a**) A tree branch mistaken as a target. (**b**) The bounding box partly overlapped with the target.

Since the segmentation index AP75 of the YOLACT++ model was 100%, we only analyzed the false and missed detection of ID tags by EfficientDet-D4. After statistics were compiled, there were no missing ID tags. False detection mainly included two cases: (1) a tree branch in the background was mistaken for the target, and the confidence was slightly high, as shown in Figure 13a; (2) a bounding box that overlapped with the tag but did not contain the numbers on the tag completely, as shown in Figure 13b. The reason for the first type of false detection may be that the high-level semantic features of the branches in this region were coincidentally similar to the ID tag, which led to the misjudgment of the branches as the target by the model. The reason for the second kind of misdetection may be that part of the ID tag was also included in the bounding boxes of these false detections, which led to the network failing to make correct judgements. Alternatively, these false bounding boxes were not filtered out when NMS was carried out. The confidence of false bounding boxes was generally low and could have been filtered by setting a confidence threshold.

### 4.5. ID Number Recognition

**Figure 14:**
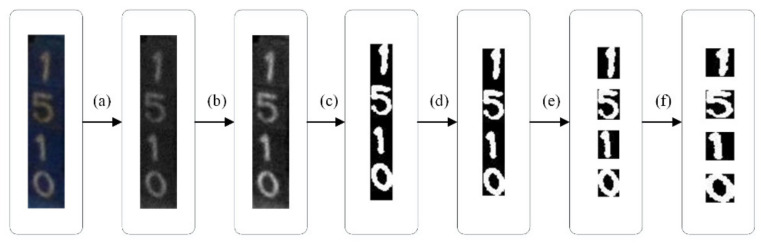
The process of character segmentation: (**a**) graying; (**b**) grey-level transformation; (**c**) binary segmentation; (**d**) morphological processing and removal of redundant background; (**e**) character cropping; (**f**) character normalization.

ID number recognition is completed by character segmentation and character recognition. The purpose of character segmentation is to segment the color tag image into four binary images containing only a single character, which was implemented through the following steps (as shown in Figure 14). The character recognition model was constructed based on a simple convolutional neural network, with the purpose to classify the single character images. The unsegmented images detected by the EfficientDet-D4 model and the segmented images by the cascaded model proposed in this paper were passed through character segmentation model and character recognition model, respectively. The character segmentation model consists of several simple image processing methods, which are illustrated in Figure 14. The character recognition model is constructed based on LeNet-5 [34]. We changed the C5 layer of LeNet-5 from fully connected layer to convolution layer to obtain the character recognition model. The reason for this change is to reduce the redundant parameters and enrich the features.

**Figure 15:**
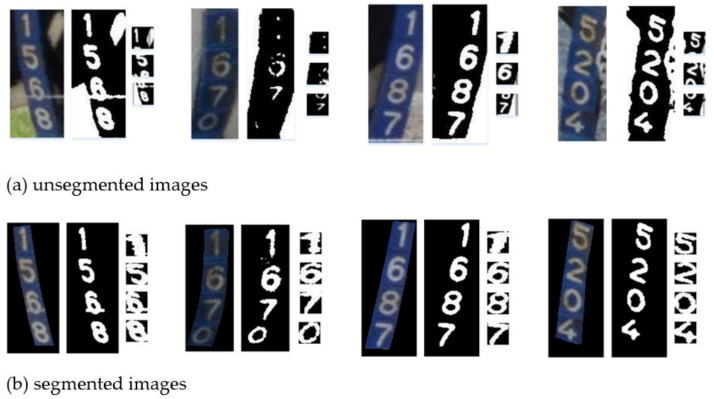
Character segmentation results of (**a**) unsegmented and (**b**) segmented images.

Table 2 shows that about 76% of ID tags in the dataset are rotated and twisted, so the corresponding detected bounding box will contain different background areas. In the binarization of pixels, the pale white body area of cows and grass in the background are misjudged as characters, which considerably interferes with the implementation of subsequent steps. If the brightness of the background exceeds that of the character, some characters will be lost due to the high threshold in the binary segmentation. Figure 15a depicts the character segmentation results of partially unsegmented images, and Figure 15b displays the character segmentation result of partially segmented images. In Figure 15, the images of each group from left to right are the images after detection/segmentation, the images after binarization, and the images after character normalization. It can be seen from the figure that the character segmentation results of the unsegmented images are very poor due to the influence of the background in the detection bounding box. Its character recognition result is obviously lower than that of segmented images. The accuracy of the character recognition of the segmented image is 95.4%, which is 2.05% higher than that in [22]. This proves that the segmentation of the tags image to remove the redundant background can effectively strengthen character recognition.

### 4.6. Future Studies

Although the cascaded model based on EfficientDet-D4 and YOLACT++ can achieve 96.5% segmentation accuracy, there is still room for improvement. For the false detection bounding boxes that overlap with the target but do not fully contain the target, their union set can be calculated as the detection results of the tag area, then the segmentation model can be used to remove the redundant background in the detection results. Alternatively, these false positives can be suppressed by better NMS methods, such as Fast NMS [35], which creates the highest confidence bounding box through mutual suppression of all detection boxes. Compared with the traditional method, it allows already-removed detections to suppress other detections, and less time is required.

The detection speed of the cascaded model also needs to be improved. The detection time is mainly consumed in the detection process of EfficientDet-D4. Due to the high resolution of the image, it is necessary to generate many anchors at different scales and classify and regress them, which requires considerable time. In practical applications, if we know that the size of the tag is within a certain range, the number of anchors generated on each grid point can be reduced, thus effectively simplifying the detection process. Additionally, the image contains many extra background data. If the target appears only in a specified space range, adding a spatial attention mechanism to EfficientDet can cause the network to pay more attention to the areas where the ID tag may appear. This would reduce the time required to extract features from irrelevant backgrounds, thus improving the detection efficiency.

## 5. Conclusions

This paper proposed a cascaded method for the instance segmentation of a cow collar ID tag based on EfficientDet-D4 and YOLACT++, which accurately detects and segments the target with a small area. The detection accuracy AP50 of the EfficientDet-D4 model is 96.5%, the segmentation accuracy AP75 of the YOLACT++ model is 100%, and the overall segmentation accuracy is 96.5%. Compared with common instance segmentation models, the accuracy is improved by more than 11.2%. Changes in brightness and deformation of the tag have little effect on the detection accuracy of the proposed model. It shows high anti-interference capability and has the potential to be applied to remote and multi-target cow identification on dairy farms. In the future, we can optimize the structure of EfficientDet and propose a better NMS method to reduce the false detection. Additionally, an attention mechanism and other strategies can be considered for reducing the time used by the feature extraction process to improve the detection speed when an image has a large background area.

## Figures and Tables

**Table 1:** The precision and efficiency of cascaded model and other models.

Model		Backbone	AP75 (%)	Inference Time (s/Iteration)
Cascaded model	EfficientDet-D4	EfficientNet-B4	96.5	1.92
	YOLACT++	ResNet50		
Mask RCNN		ResNet101	85.3	2.63
Mask Scoring RCNN		ResNet101	58.2	3.39
SOLOv2		ResNet101	18.5	1.21

**Table 2:** ID tags detection accuracy with different types of deformation.

State of the Tag	The Number of Images	AP50 (%)
Slight rotation and distortion	94	99.4
Rotation	192	97.8
Rotation and distortion	120	96.5

## Data Availability

Not applicable.

## Appendix A

**Table A1:** Comparison of different identification methods for cows.

Method		Technology/Feature	Strengths	Weaknesses
RFID		Wireless transmission technology	Real time; reliable; high practicality	High costs; affected by electromagnetic environments
Machine vision	Based on biological characteristics of cows	Head region of cows (muzzle, iris, and face)	Strong distinguishing feature	Difficulties in head image acquisition; too many output categories
		Black and white patterns on the cows’ bodies	Easy to capture; no effect on cow’s activity	Only for cows with pattern; too many output categories
	Based on ID number on the tag	Ear tag	Accommodate up to more cows	Require holing on ear; Recognition angle and distance are limited
		Collar ID tag (our method)	Accommodate up to more cows	The deformation of the ID tag
